# Effect of Puerarin Regulated mTOR Signaling Pathway in Experimental Liver Injury

**DOI:** 10.3389/fphar.2018.01165

**Published:** 2018-10-23

**Authors:** Bu-Gao Zhou, Hai-Mei Zhao, Xiu-Yun Lu, Wen Zhou, Fu-Chun Liu, Xue-Ke Liu, Duan-Yong Liu

**Affiliations:** ^1^Science and Technology College, Jiangxi University of Traditional Chinese Medicine, Nanchang, China; ^2^School of Basic Medical Sciences, Jiangxi University of Traditional Chinese Medicine, Nanchang, China; ^3^Department of Postgraduate, Jiangxi University of Traditional Chinese Medicine, Nanchang, China; ^4^Key Laboratory of Pharmacology of Traditional Chinese Medicine in Jiangxi, Nanchang, China

**Keywords:** puerarin, liver injury, mTOR signaling pathway, hepatoprotective effect, apoptosis

## Abstract

It is known that excessive hepatocellular apoptosis is a typical characteristic of hepatic disease, and is regulated by the mammalian target of rapamycin (mTOR) signaling pathway. As the main active component of Kudzu (*Pueraria lobata*) roots, which is frequently used to treat hepatic diseases, Puerarin (Pue) has been reported to alleviate and protect against hepatic injury. However, it is unclear whether Pue can inhibit mTOR signaling to prevent excessive apoptosis in the treatment of hepatic diseases. In the present study, Pue effectively ameliorated pathological injury of the liver, decreased serum enzyme (ALT, AST, γ-GT, AKP, DBIL, and TBIL) levels, regulated the balance between pro-inflammatory (TNF-α, IL-1β, IL-4, IL-6, and TGF-β1) and anti-inflammatory cytokines (IL-10), restored the cell cycle and inhibited hepatocellular apoptosis and caspase-3 expression in rats with liver injury induced by 2-AAF/PH. Pue inhibited p-mTOR, p-AKT and Raptor activity, and increased Rictor expression in the liver tissues of rats with experimental liver injury. These results indicated that Pue effectively regulated the activation of mTOR signaling pathway in the therapeutic and prophylactic process of Pue on experimental liver injury.

## Introduction

Hepatopathy is a common and frequently occurring disease, which has severe adverse consequence**s** in patients and is a heavy financial burden in China. The pathogenesis of liver injury is extremely complicated; however, it is known that the mammalian target of rapamycin (mTOR) signaling pathway plays an important role in the development of liver injury. The pathways related to mTOR signaling in the pathogenesis of hepatopathies include the LKB1/AMPK/mTOR metabolic axis, PI3K/AKT/mTOR and Bcl-2/Bax-mediated apoptosis/autophagy, and ERK/mTOR-mediated autophagy, the AKT/mTOR/c-Myc axis, NF-κB/mTOR signaling and TSC/mTOR signaling (Bhangoo et al., 2017; Chang et al., 2017; Li et al., 2017; Wang et al., 2017; Hu et al., 2018; Majd et al., 2018; Wei et al., 2018).

Puerarin (Pue) is a natural flavonoid compound extracted from the traditional Chinese herb *Radix puerariae*, which has been used to treat alcoholic disease in traditional Chinese medicine for more than a millennium (Chen et al., 2013; Li et al., 2013; Peng et al., 2013). In Chinese pharmacopeia (CP) 2010, second section, its chemical formula and molecular weight is C_21_H_20_O_9_, and 416.37800, respectively. And the other physico-chemical properties and chromatogram of Pue are listed in the CP. Puerarin possesses various pharmacological activities, including anti-oxidant, anti-inflammatory, antiapoptosis, cardioprotective, anti-cancer, hepatoprotective, and anti-diabetic properties. It has been shown to have a satisfactory therapeutic effect in many diseases such as hepatopathy, alcohol-related disorders and Parkinson’s disease in both animal and clinical studies (Li et al., 2014; Han et al., 2015; Jiang et al., 2016; Li et al., 2016; Xu et al., 2016; Yang et al., 2016). Hepatopathies treated with Pue include liver injury due to non-alcoholic fatty liver disease, liver ischemia/reperfusion injury, chronic and acute alcohol-induced liver injury, concanavalin A-induced liver injury, liver fibrosis induced by CCl4 and LPS/D-Gal-induced acute liver injury (Guo et al., 2013; Mahdy et al., 2014; Jiang et al., 2016; Zhao W. et al., 2016; Li et al., 2018; Xiao et al., 2018).

In previous studies, Pue improved liver histopathology, reduced plasma alanine aminotransferase (ALT) and aspartate aminotransferase (AST) levels, reduced the production of pro-inflammatory cytokines, and suppressed hepatocyte apoptosis to protect against liver injury, which was achieved by restoring autophagy, modulating the TLR4 or GSK-3β/NF-κB pathway, JNK/c-Jun/CYP7A1 pathway, TLR4/p38/CREB pathway, regulating metabolic function and inhibiting PARP-1 and insulin receptors (Li et al., 2013; Ma et al., 2014; Liu et al., 2016; Zhao L. et al., 2016; Li et al., 2018; Xiao et al., 2018). Interestingly, Pue restored the impaired autophagic flux in Pb-treated proximal tubular cells partly by activating autophagy via AMPK/mTOR signaling pathway (Song et al., 2017). However, it is not known if Pue can regulate the mTOR signaling pathway to protect against liver injury. The increasing evidences had indicated that 2-AAF may possess a mechanism that is analogous to a wide variety of hepatotoxins (Oh et al., 2007). An exposure to 2-AAF can lead to hepatocyte death and destruction of cellular structures and functions in animals. Previous studies have illustrated that the 2-AAF/PH animal model of liver injury is one of the most widely used models of liver diseases to explore the mechanism of action and therapeutic effect of various medicines (Zheng et al., 2006; Bae et al., 2011). In the present study, in order to determine the mechanism of Pue in the treatment of liver injury, we analyzed its curative effect and the mTOR signaling pathway in liver injury induced by 2-acetylaminofluorene (2AAF)/partial hepatectomy (PH) in rats.

## Materials and Methods

### Animal Experiments

The animal experiments in the present study were approved by the Animal Ethics Committee of Jiangxi University of Traditional Chinese Medicine (JXUTCM). All animals were handled in accordance with the guidelines on animal welfare according to the Institutional Animal Care and Use Committee (IACUC) of JXUTCM. The protocol (permit number: JZ2017-136) of the present study was approved by the IACUC.

### Animal Preparation

**FIGURE 1:**
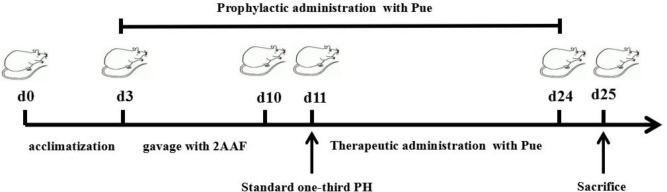
The flow diagram of experimental operation.

Fifty male Wistar rats were supplied by the Animal Center of Peking University Health Science Center (animal certificate number SCXK 2006-0008). Rats with a body weight of 250–350 g were included in the study. These animals were randomly divided into 5 groups: normal (Normal), 2-AAF/PH model (Model), 2-AAF/PH rats treated with prophylactic administration of Pue (PueP), which was performed at the same time with the gavage of 2-AAF, 2-AAF/PH rats treated with therapeutic administration of Pue (PueT), and 2-AAF/PH rats treated with rapamycin (RAPA), which was performed on the second day after PH. Each group comprised ten rats. They were bred in the JXUTCM Animal Facility with free access to a standard diet and tap water under a 12-h light/dark cycle and constant room temperature (25 C). The rats were acclimatized to these conditions for 3 days prior to the experimental studies. The flow diagram of experimental operation was shown in the Figure 1.

### 2-AAF/PH-Induced Liver Injury

According to previous reports by Shiota et al. (2000) and Oh et al. (2007), with the exception of rats in the Normal group, rats in the other three groups were administered 10 mg/kg of 0.2% 2-AAF by gavage for 7 days. Rats in the Normal group received physiological saline solution. Following 7 days administration of 2-AAF or physiological saline solution, anesthesia was initiated by an intraperitoneal injection of sodium pentobarbital in all animals. Standard one-third PH was performed in rats from the Model, PueP, PueT, and RAPA groups. In the Normal group, the abdomens of rats were opened along with the ventrimeson, and 1 mL peripheral blood was collected from the portal vein.

### Drugs

Pue injection (batch number 1301157, purity: 110.06 g⋅L^−1^, by HPLC) was purchased from Shandong Fangming Pharmaceutical Co., Ltd. (Heze, China). 2-AAF was purchased from Sigma (St. Louis, MO, United States). Rapamycin (batch no. 20150483) was from Wyeth Pharmaceuticals Company (Philadelphia, PA, United States).

### Treatment Protocol

In the PueP group, at the same time as 2-AAF administration, the rats were administered 200 mg/kg Pue by intraperitoneal (IP) injection until sacrifice (The prophylactic administration of Pue was temporarily stopped 1 day for PH). In the PueT and RAPA groups, rats were administered 200 mg/kg Pue or 0.2 mg/kg RAPA, respectively, by IP injection for 14 days. Rats in the Normal and Model groups were administered the same volume of physiological saline. On the 15th day after PH, all animals were anesthetized with 10% urethane and sacrificed. Liver tissues were separated and liver weight was measured. Some liver tissues were immediately immersed in liquid nitrogen and kept in a freezer at −80 C until analysis. From some of the remaining fresh liver tissues, a cell suspension was prepared for flow cytometry analysis.

### Analysis of Serum Enzymes

Serum enzyme levels are important indices in the evaluation of liver function. These enzymes include serum albumin, alanine aminotransferase (ALT), AST, gamma-glutamyl transpeptidase (γ-GT), alkaline phosphatase (AKP), direct bilirubin (DBIL), and total bilirubin (TBIL). Peripheral blood samples (*n* = 8) were collected from the aorta and the serum was separated by centrifugation at 500 × *g* for 15 min. The enzymes in serum were assayed using an automatic biochemical analyzer.

### Pathological Observations

Liver tissues were fixed in 4% paraformaldehyde for 7–10 days, embedded in paraffin, and then cut into sections at 8-μm thickness. All sections were stained with Hematoxylin and Eosin (H&E) and observed by light microscopy.

### Liver Supernatant Preparation and Quantitative Analysis of Proteins

Liver tissues (*n* = 8) were lysed in RIPA buffer with protease and phosphate inhibitor cocktail (Merck, Ashland, MA, United States) using a sonicator. Crude lysates were centrifuged at 20,000 × *g* for 20 min at 4°C. The liver supernatant was collected for further analysis. Protein concentrations (*n* = 8) in the liver supernatants were determined using the classic BCA protein assay (Beyotime, Nanjing, China).

### Cytokine Expression Analyzed by ELISA

The expression of TNF-α, IL-1β, IL-4, IL-6, IL-10, and TGF-β1 in liver supernatant was analyzed by enzyme-linked immunosorbent assay (ELISA). All ELISA operational steps were performed according to commercial ELISA kits (eBioscience, San Diego, CA, United States). The absorbance at 450 nm was read using a microplate reader (Bio-Rad, Hemel Hempstead, United Kingdom).

### Bromodeoxyuridine (BrdU) Staining and Flow Cytometry (FCM)

According to the BD Pharmingen BrdU flow kit instruction manual (No. 557891) (BD Company, San Diego, CA, United States), all rats (*n* = 8) in the five groups were injected IP with 2 mg/kg BrdU solution, which was prepared using a 10 mg/mL solution of BrdU in sterile 1 × DPBS. The rats were executed 1 h after injection. Hepatocytes were isolated, resuspended, and successively incubated with Cytofix/Cytoperm buffer for 30 min, Cytoperm permeabilization for 10 min, and then 300 mg/mL/d DNase in DPBS. At 1 h after incubation with DNase, the above cells were incubated with 50 mL FITC-anti-BrdU for 20 min at room temperature, and then with 20 mL 7-AAD in 1 mL staining buffer for 30 min in the dark, and were finally analyzed by flow cytometry (FACSCalibur; BD Company).

### Caspase-3 Level Assayed by FCM

Hepatocytes (*n* = 8) were separated from fresh liver tissues, and fixed in Fix/Perm Buffer (eBioscience, CA, USA) for 1 h at room temperature, and then incubated with cleaved caspase-3 antibody (FITC, BD Pharmingen) for 1 h in the dark at 37 C. All stained cells were analyzed by flow cytometry.

### Western Blot Analysis

To analyze the electrophoretic mobility of mTOR, p-mTOR, AKT, p-AKT, Rictor, and Raptor, protein concentrations (*n* = 6) were determined using the classic BCA protein assay (Beyotime). Twenty μg protein from each sample was fractionated by sodium dodecyl sulfate polyacrylamide gel electrophoresis (SDS-PAGE) and transferred onto a polyvinylidene fluoride (PVDF) membrane using Bio-Rad Western blot apparatus. After the membranes were blocked, they were incubated with the primary antibody [GAPDH (1:2000), mTOR (1:2000), phosphor (p)-mTOR (1:1000), AKT (1:2000), p-AKT (1:2000), Rictor (1:3000), or Raptor (1:1000)] for 12 h at 4°C. The next day, the membrane was incubated with the HRP secondary antibody (goat anti-rat, 1:4000) for 1 h. The blots were visualized by enhanced chemiluminescence, and detected via a Fuji LAS4000 imager and quantified by Quantity One 4.40 software (Bio-Rad, CA, United States).

### Statistical Analysis

All data in the present study were analyzed using Prism 4.0 (GraphPad Software, CA, United States), and expressed as the mean ± standard error of the mean (SEM). One-way ANOVA was used to assess the differences between the means of the groups followed by Tukey’s *post hoc* test. Significance was accepted at *p* < 0.05.

## Results

### Puerarin Ameliorated Pathological Changes in Rats With 2-AAF/PH-Induced Liver Injury

**FIGURE 2:**
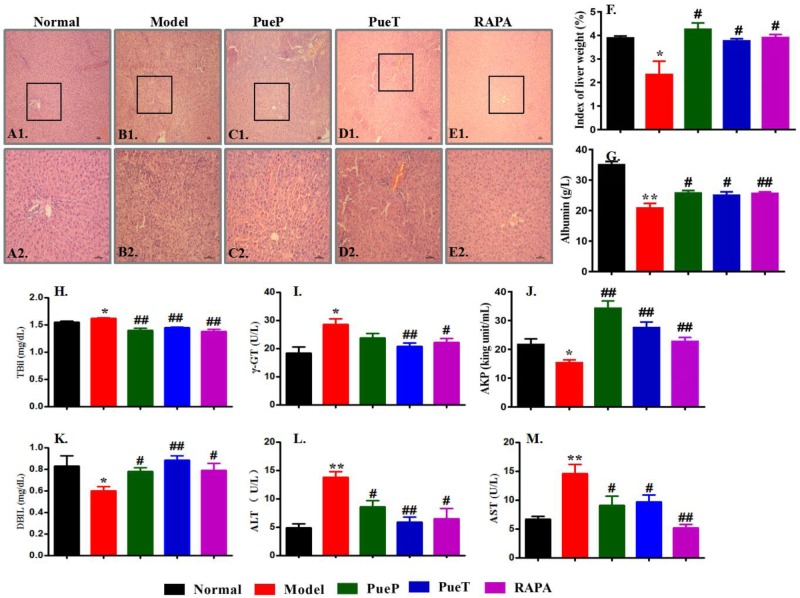
Typical pathological changes and levels of serum enzymes in liver injury induced by 2-AAF/PH. Liver histology in the Normal group [**(A1,A2)**], Model group (**(B1,B2)**], PueP group [**(C1,C2)**], PueT group [**(D1,D2)**], and RAPA group [**(E1,E2)**]; liver tissues were stained with hematoxylin and eosin. **(A1–E1)**: Bar = 100 μm, **(A2–E2)**: Bar = 200 μm. The liver index was measured as liver weight (g)/body weight (g) × 100%. **(F)**: Liver weight; biochemical parameters in blood related to liver function in rats administered Pue after 2-AAF treatment combined with PH. These parameters included albumin **(G)**, TBIL **(H)**, γ-GT **(I)**, AKP **(J)**, DBIL **(K)**, ALT **(L)**, and AST **(M)**. Data were expressed as mean ± standard error of the mean (*n* = 8).^∗^*p* < 0.05 and ^∗∗^*p* < 0.01 versus Normal group; ^#^*p* < 0.05 and ^##^*p* < 0.01 versus Model group.

Typical pathological characteristics of liver injury were seen in the liver tissues of rats in the Model group (Figures 2B1,B2), and included structural disorder of hepatic lobules, mass hepatocyte necrosis, hepatocyte fatty degeneration, physaliphora formation, inflammatory cell infiltration, and tissue edema. These typical pathological characteristics were found in untreated rats with liver injury induced by 2-AAF/PH and an decrease in the index of liver weight was also observed (Figure 2F), compared with the Normal control group (Figures 2A1,A2,B1,B2,F). In addition, compared with rats in the Model group, all pathological characteristics of liver injury were markedly ameliorated in rats with experimental liver injury treated prophylactically or therapeutically with Pue or RAPA, together with a marked increase in the index of liver weight (Figures 2B1,B2,C1,C2,D1,D2,E1,E2,F). These results showed that Pue effectively ameliorated pathological liver injury induced by 2-AAF/PH.

Serum levels of enzyme activity are important biochemical markers of liver failure, and can confirm the protective effect of Pue in liver injury induced by 2-AAF/PH. As shown in Figures 2H–M, serum ALT (Figure 2L), AST (Figure 2M), γ-GT (Figure 2I) and TBIL (Figure 2H) levels were markedly increased in the Model group compared with the Normal, PueP, PueT, and RAPA groups. However, the levels of albumin (Figure 2G), serum AKP (Figure 2J), and DBIL (Figure 2K) were significantly reduced in the Model group compared with the Normal, PueP, PueT and RAPA groups. These results demonstrated that Pue decreased serum enzyme levels (such as ALT and AST) to prevent liver injury induced by 2-AAF/PH.

### Puerarin Regulated Cytokine Expression in the Liver Tissues of Rats With 2-AAF/PH-Induced Liver Injury

**FIGURE 3:**
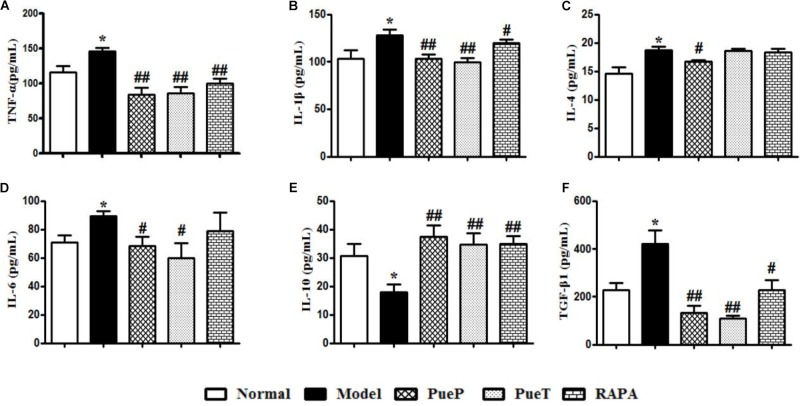
ELISA analysis of related inflammatory cytokines and TGF-β1 in serum. Inflammatory cytokines and TGF-β1 play important roles in the inflammatory reaction and hepatic fibrosis to induce liver diseases. TNF-α **(A)**, IL-1β **(B)**, IL-4 **(C)**, IL-6 **(D)**, IL-10 **(E)**, and TGF-β1 **(F)**. Data are expressed as mean ± SEM (*n* = 8).^∗^*p* < 0.05 and ^∗∗^*p* < 0.01 versus Normal group; ^#^*p* < 0.05 and ^##^*p* < 0.01 versus Model group.

An imbalance in cytokines plays an important role in the pathogenesis of liver injury. In the present study, hepatic TNF-α (Figure 3A), IL-1β (Figure 3B), IL-4 (Figure 3C), IL-6 (Figure 3D), and TGF-β1 (Figure 3F) expression was significantly increased in untreated rats with experimental liver injury compared with the Normal group, while IL-10 (Figure 3E) expression decreased significantly. However, the levels of hepatic TNF-α (Figure 3A), IL-1β (Figure 3B), IL-4 (Figure 3C), IL-6 (Figure 3D), and TGF-β1 (Figure 3F) were significantly lower in rats with 2-AAF/PH-induced liver injury treated with Pue and RAPA than in untreated rats with liver injury, and IL-10 expression was higher (Figure 3E). These results demonstrated that Pue regulated the cytokine balance to inhibit inflammatory injury of the liver.

### Puerarin Controlled the Cell Cycle and Caspase-3 Expression in Hepatocytes of Rats With 2-AAF/PH-Induced Liver Injury

**FIGURE 4:**
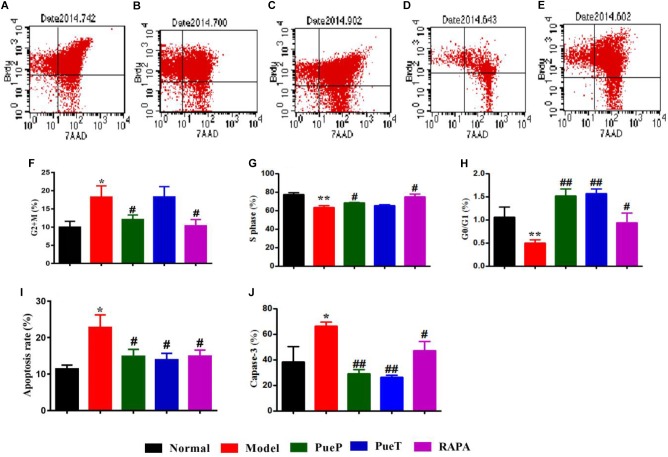
BrdU staining and caspase-3 expression in hepatocytes from rats with liver injury induced by 2AAF/PH. Cellular incorporation of BrdU staining can be detected by flow cytometry to analyze apoptosis and proliferation *in vivo*. Hepatocytes in rats with liver injury induced by 2AAF/PH were labeled following an intraperitoneal injection of BrdU, and BrdU staining was measured by flow cytometry, anti-BrdU antibody and 7-AAD. Representative scatter diagrams of each group are shown: Normal group **(A)**, Model group **(B)**, PueP group **(C)**, PueT group **(D)**, and RAPA group **(E)**. Results were analyzed by Flowjo version 7.6.1 software, and included the number of cells in G2 + M phase **(F)**, S phase **(G)**, G0/G1 phase **(H)**, and apoptotic cells **(I)**. Caspase-3 expression **(J)**. Data are expressed as mean ± SEM (*n* = 8).^∗^*p* < 0.05 and ^∗∗^*p* < 0.01 versus Normal group; ^#^*p* < 0.05 and ^##^*p* < 0.01 versus Model group.

BrdU staining is a traditional method for evaluating the changes in cell cycle and apoptosis. Caspase-3 is a biological marker of early phase apoptosis. As shown in Figures 4A–E,H, the number of liver cells in G0/G1 phase in untreated rats with experimental liver injury was lower than that in rats with 2-AAF/PH-induced liver injury treated with PueT, PueP, and RAPA. In the S phase, with the exception of rats in the PueP group, the number of hepatocytes in the PueT and RAPA groups was higher than that in the Model group (Figures 4A–E,G). In addition, the number of hepatocytes in rats with liver injury pre-treated with Pue and treated with RAPA was markedly decreased compared with untreated rats with experimental liver injury (Figures 4A–E,G). Hepatocyte apoptosis level and caspase-3 expression were markedly increased in untreated rats with 2-AAF/PH induced liver injury compared with the Normal group (Figures 4A–E,I). In addition, hepatocyte apoptosis level and caspase-3 expression in rats with liver injury in the PueP, PueT and RAPA groups were decreased compared with the Model group (Figures 4A–E,I,J). These results illustrated that Pue down-regulated excessive apoptosis by inhibiting over-expression of caspase-3 and recovering hepatocytes in liver injury induced by 2-AAF/PH.

### Puerarin Inhibited the mTOR Signaling Pathway in Liver Tissues of Rats With 2-AAF/PH-Induced Liver Injury

**FIGURE 5:**
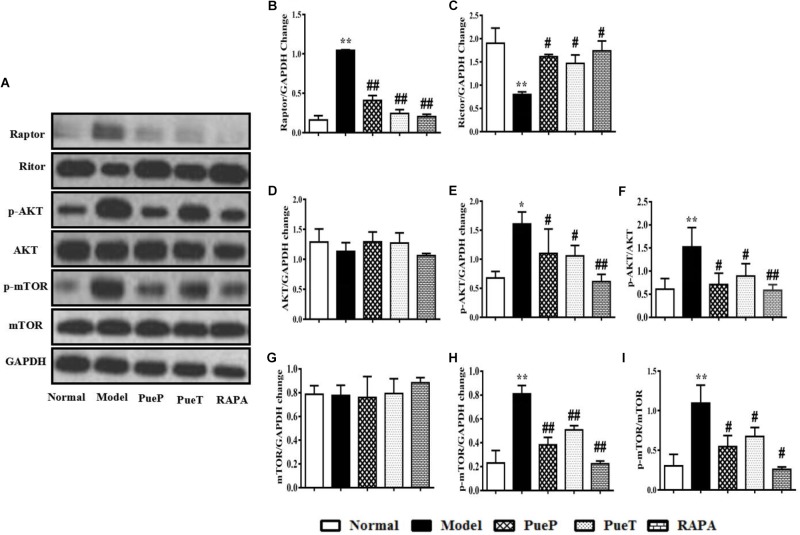
Western blotting of mTOR, p-mTOR, AKT, p-AKT, Raptor and Rictor. **(A)** Western blotting of mTOR, p-mTOR, AKT, p-AKT, Raptor and Rictor. **(B)** Quantitative analysis of Raptor. **(C)** Quantitative analysis of Rictor. **(D)** Quantitative analysis of AKT. **(E)** Quantitative analysis of p-AKT. **(F)** Ratio of p-Akt/Akt. **(G)** Quantitative analysis of mTOR. **(H)** Quantitative analysis of p-mTOR. **(I)** Ratio of p-mTOR/mTOR. Data are presented as mean ± SEM (*n* = 3). ^∗^*p* < 0.05 and ^∗∗^*p* < 0.01 versus Normal group; ^#^*p* < 0.05 and ^##^*p* < 0.01 versus Model group.

mTOR signaling is a pivotal pathway in the regulation of cell growth, development and apoptosis. In the present study, we observed the changes in mTOR, and its upstream and downstream proteins by Western blot analysis. As shown in Figure 5, p-mTOR (Figure 5A,G,H) and p-AKT (Figures 5A,D,E) protein expression was markedly up-regulated in the liver tissues of untreated rats with experimental liver injury compared with the Normal group, and a similar trend in the ratio of p-mTOR/mTOR (Figure 5I) and p-AKT/AKT was also observed (Figure 4F). Interestingly, in contrast to the Model group, p-mTOR (Figure 5A,G,H) and p-AKT (Figures 5A,D,E) protein expression was inhibited, and the ratio of p-mTOR/mTOR (Figure 5I) and p-AKT/AKT (Figure 5F) were reduced in rats with 2-AAF/PH-induced liver injury treated or pretreated with Pue and RAPA. As important protein molecules in the mTOR signaling pathway, the over-expression of Raptor (Figures 5A,B) and down-regulated expression of Rictor (Figures 5A,C) were synchronously observed in the Model group in the present study. However, following administration of Pue and RAPA, Raptor (Figures 5A,B) expression was inhibited, and Rictor (Figures 5A,C) expression was activated in the PueP, PueT, and RAPA groups compared with the Model group. These results showed that Pue inhibited activation of the mTOR signaling pathway in 2-AAF/PH-induced liver injury.

## Discussion

As the main active component in Kudzu (*P. lobata*) roots, which is frequently used to treat hepatic disease, Pue has been reported to alleviate and protect against hepatic injury. Increased researches had indicated that Pue effectively protected against the varied liver injury diseases via multiple pathway including anti-oxidant, anti-inflammatory, antiapoptosis, and inhibiting inflammatory factors expression. However, its mechanism is unclear (Li et al., 2014; Han et al., 2015; Jiang et al., 2016; Li et al., 2016; Xu et al., 2016; Yang et al., 2016). In the present study, Pue effectively ameliorated pathological liver injury, decreased serum enzymes, restored the cytokine balance, limited caspase-3 activity and controlled the cell cycle in hepatocytes to inhibit excessive apoptosis. Song and his workmates found that Pue can act as an antioxidant to inhibit proximal tubular cell apoptosis by restoring mitochondrial function, up-regulating Bcl-2 and down-regulating of Bax and increasing Bcl-2/Bax ratio (Song et al., 2016). Our results were kept similarity with the Song’s reports. This evidence suggests that the hepatoprotective effect of Pue in the treatment and prevention of hepatic disease is closely related to its antiapoptosis activity.

Excessive apoptosis of hepatocytes plays a significant role in the course of hepatic disease and leads to pathological liver injury, liver dysfunction, fibrosis/cirrhosis, and tumorigenesis (Ryu and Han, 2011; Wang and Lin, 2013; Wang, 2014, 2015). The previous studies had indicated that activated apoptosis-related proteins accelerate G1/S cell cycle transition and result in apoptosis and cell death (Iiboshi et al., 1999; Ryu and Han, 2011). In the present study, the apoptosis rate and the percentage of cells in G2 phase, and expression of caspase-3 were increased in the experimental liver injury rats. Thus, inhibiting excessive apoptosis via various pathways is considered to be an important therapeutic strategy in liver disease.

In the many pathways regulating hepatocellular apoptosis, mTOR may also have a pleiotropic function in the regulation of apoptosis (Velagapudi et al., 2011). mTOR is a serine/threonine kinase that modulates several important aspects of mammalian cell function, including the initiation of protein translation, cell proliferation, mortality, and survival protein synthesis (Duman et al., 2012; Malik et al., 2013), and nucleates mTOR complex 1 (mTORC1) and mTOR complex 2 (mTORC2). mTORC1 has five components including mTOR, regulatory-associated protein of mTOR (Raptor), target of rapamycin complex subunit LST8 (mLST8), proline-rich AKT1 substrate 1 (PRAS40), and DEP domain-containing mTOR-interacting protein (DEPTOR), while mTORC2 has six components, including mTOR, RPTOR-independent companion of mTOR (Rictor), target of rapamycin complex 2 subunit MAPKAP1 (mSIN1), protein observed with Rictor-1 (Protor-1), mLST8, and DEPTOR6 (Gao et al., 2002). The mTOR signaling pathway has a pleiotropic function in the regulation of apoptosis. Recently, a number of reports have shown that the mTOR pathway plays a significant role in activation of the apoptotic signaling cascade (Velagapudi et al., 2011). Upstream of the mTOR signaling pathway, activated PI3K/AKT can in turn phosphorylate and activate Raptor and Rictor. As a classic mechanism of apoptosis, highly expressed Raptor can activate eukaryotic translation initiation factor 4E-binding protein 1 and p70s6k to inhibit Rictor, and can increase the activity of many apoptosis-related proteins to accelerate cell cycle transition, leading to apoptosis and cell death (Iiboshi et al., 1999; Ryu and Han, 2011). In the present study, Western blot analyses found that p-mTOR, p-AKT, and Raptor were highly expressed and activated, while Rictor activity was inhibited in the liver tissue of rats with 2-AAF/PH-induced liver injury. According to the above-mentioned results, as excess hepatocellular apoptosis was observed in experimental liver injury, we suggest that the overactive mTOR signaling pathway induced excessive hepatocellular apoptosis, leading to pathological structural damage, and finally resulted in liver injury induced by 2-AAF/PH. Prophylactic and therapeutic treatment with Pue in rats with experimental liver injury effectively inhibited p-mTOR, p-AKT, and Raptor expression and activated Rictor to reduce hepatocellular apoptosis, and ameliorated pathological injury of liver tissues. These findings suggest that the protective effect of Pue in hepatic disease is closely related to inhibition of the mTOR signaling pathway via inactivation of AKT protein.

In summary, Pue effectively regulated the activation of mTOR signaling pathway in the therapeutic and prophylactic process of Pue on experimental liver injury. Our results hinted that protective effect of Pue on the 2-AAF/PH induced liver injury were realized by inhibiting mTOR signaling pathway, which will provide an inportant idea to explore the target of Pue protected against liver disease. It is known that mTOR signaling pathway plays a significant role in the process of autophagy. However, it is still unclear whether Pue regulates hepatocellular autophagy by regulating mTOR and related genes, we will add the *in vitro* hepatic cell study with SiRNA for mTOR, which will be an extremely valuable topic of research in our next study.

## Author Contributions

H-MZ and B-GZ contributed equally to this work as joint first authors and designed and performed the research. X-YL and WZ contributed to analysis of data, as well as writing and reviewing the final manuscript. B-GZ, H-MZ, X-YL, WZ, F-CL, and X-KL performed the research. D-YL was involved in project conception, design, and data analysis, as well as writing and reviewing the final manuscript.

## Conflict of Interest Statement

The authors declare that the research was conducted in the absence of any commercial or financial relationships that could be construed as a potential conflict of interest.
